# A Pilot Study of Blood-Based Methylation Markers Associated With Pancreatic Cancer

**DOI:** 10.3389/fgene.2022.849839

**Published:** 2022-03-14

**Authors:** Rick J. Jansen, Megan Orr, William R. Bamlet, Gloria M. Petersen

**Affiliations:** ^1^ Department of Public Health, North Dakota State University, Fargo, ND, United States; ^2^ Genomics, Phenomics, and Bioinformatics Program, North Dakota State University, Fargo, ND, United States; ^3^ Center for Immunization Research and Education (CIRE), North Dakota State University, Fargo, ND, United States; ^4^ Center for Diagnostic and Therapeutic Strategies in Pancreatic Cancer, North Dakota State University, Fargo, ND, United States; ^5^ Department of Statistics, North Dakota State University, Fargo, ND, United States; ^6^ Department of Quantitative Health Sciences, Mayo Clinic, Rochester, MN, United States

**Keywords:** pancreatic cancer, methylation, age predictor, biomarker, gene expression, lymphocyte

## Abstract

Over the past several decades in the United States, incidence of pancreatic cancer (PCa) has increased, with the 5-year survival rate remaining extremely low at 10.8%. Typically, PCa is diagnosed at an advanced stage, with the consequence that there is more tumor heterogeneity and increased probability that more cells are resistant to treatments. Risk factors for PCa can serve as a way to select a high-risk population and develop biomarkers to improve early detection and treatment. We focus on blood-based methylation as an approach to identify a marker set that can be obtained in a minimally invasive way (through peripheral blood) and could be applied to a high-risk subpopulation [those with recent onset type 2 diabetes (DM)]. Blood samples were collected from 30 patients, 15 had been diagnosed with PCa and 15 had been diagnosed with recent onset DM. HumanMethylationEPIC Beadchip (Illumina, CA, United States) was used to quantify methylation of approximately 850,000 methylation sites across the genome and to analyze methylation markers associated with PCa or DM or both. Exploratory analysis conducted to propose importance of top CpG (5′—C—phosphate—G—3′) methylation site associated genes and visualized using boxplots. A methylation-based age predictor was also investigated for ability to distinguish disease groups from controls. No methylation markers were observed to be significantly associated with PCa or new onset diabetes compared with control the respective control groups. In our exploratory analysis, one methylation marker, CpG04969764, found in the Laminin Subunit Alpha 5 (*LAMA5*) gene region was observed in both PCa and DM Top 100 methylation marker sets. Modification of *LAMA5* methylation or *LAMA5* gene function may be a way to distinguish those recent DM cases with and without PCa, however, additional studies with larger sample sizes and different study types (e.g., cohort) will be needed to test this hypothesis.

## Introduction

Development of effective methods to treat pancreatic cancer (PCa) has eluded investigators. Over the past several decades in the United States, incidence of PCa has increased, with the 5-year survival rate remaining extremely low at 10.8% (Bengtsson et al., 2020). Typically, PCa is diagnosed at an advanced stage, and as a consequence observed molecular changes display increased tumor heterogeneity and would also define potentially a fraction of cells that are more resistant to therapeutic treatments (Chan-Seng-Yue et al., 2020; Raphael et al., 2017).

Risk factors for PCa can serve as a way to select high-risk populations for which biomarkers could be developed to improve early detection and treatment. The risk factors consistently identified as associated with PCa include cigarette smoking, age, sex, family history, longstanding type 2 diabetes mellitus (DM) or pancreatitis, and obesity (Ghadirian et al., 2003; Hassan et al., 2007; Vrieling et al., 2009). Recent onset DM (developed within 3 years prior to PCa diagnosis) has been shown to potentially be the result of the presence of the PCa tumor (Aggarwal et al., 2013; Sharma et al., 2018). Another important component of a screening program is trying to make it as minimally invasive to ensure it is acceptable and causes minimal burden to the participant. Biomarkers obtained via peripheral blood would be less invasive than those obtain via tissue from the pancreas.

Genome-wide methylation and gene expression marker profiles have been created to subtype disease, identify blood cell types and methylation markers associated with several different cancers (Johnson et al., 2021; Yu et al., 2021; Xu et al., 2021; Zhao et al., 2021). Publicly available genomic datasets have been used to identify methylation markers and genes associated with several specific risk factors important in PCa such as smoking, obesity, and diabetes (Ehrlich, 2002; Richardson, 2003; Toperoff et al., 2012). Mechanistically, variation in DNA methylation likely reflects variation in histone modifications, chromatin conformation, and gene expression, (Xu and Taylor, 2014) with hypo-methylation of the promoter region and hyper-methylation of the gene body often reflecting increased expression (Jones, 2012).

In this pilot study, we sought to determine which blood-based methylation markers warrant further exploration as biomarkers in the setting of either PCa or recent onset diabetes mellitus and PCa. We focus on blood-based methylation in order to identify a marker set that can be obtained in a minimally invasive way (through peripheral blood) and could be used in a high-risk subpopulation (those with recent DM). Exploratory analyses were conducted and public databases used to develop hypothesis for further exploration.

## Materials and Methods

### Participant Population

Participant recruitment protocols have been detailed elsewhere (Wang et al., 2007; Hu et al., 2018). Information and blood samples were collected from a total of 3,932 prospectively recruited PCa cases and 2,397 controls recruited through Mayo Clinic primary care clinics. Of these, 30 patients were identified and selected for this study. Of these 30 patients, 15 had been diagnosed with PCa and 15 without PCa had been diagnosed with recent type 2 diabetes.

### Data Collection and Measurement

The study protocol was reviewed and approved by the Mayo Clinic Institutional Review Board. All eligible individuals provided written informed consent to participate in the study. Information on demographic characteristics, body mass index (BMI), lifestyle, and comorbid conditions were collected using a self-administered questionnaire for both cases and controls.

Blood samples were collected from cases at the time of diagnosis and prior to receiving any treatment for PCa. Blood samples were collected from controls at the time of a routine medical visit. The Biospecimens, Accessioning, and Processing (BAP) core at Mayo Clinic extracted 25 ul of dsDNA at a concentration of 50 ng/ul. (Qiagen) The HumanMethylationEPIC Beadchip (Illumina, CA, United States) was used in this study to quantify methylation of approximately 850,000 methylation sites across the genome using standard protocols. To generate methylation β-values for all analyses, raw methylation data was normalized using negative control probes (Illumina GenomeStudio) and used the MethylationEPIC manifest for processing EPIC data. After standard quality control methods, 156,999 methylation markers remained for analysis.

### Statistical Analysis

Select descriptive demographics of the sampled population were compared using Fisher’s exact test and ANOVA F tests. Differential methylation analysis was conducted to identify disease-associated CpG sites and top methylation markers were characterized to identify disease-associated enrichment across the genome. Several logistic regression models were used to look for significant associations between CpGs and disease and all models adjusted for sex and age. Model 1 was between PCa and each CpG, model 2 was model 1 plus blood cell type adjustment, and model 3 was model 2 plus DM adjustment. Models 4–6 were the same as model 1–3 except involved DM as the primary disease instead of PCa. Genome-wide significance level was set at 9 × 10–8 for an EPIC array (Mansell et al., 2019). Exploratory analyses were conducted to propose importance of top CpG associated genes and visualized using boxplots. A methylation-based age predictor was also investigated for ability to distinguish disease associated disease groups from controls. Public databases, GTEx (GTEx Consortium, 2013) and GEO, (Barrett et al., 2013) were used to investigate methylation and gene expression data in other populations with larger sample sizes and as a way to provide biological context or functional importance of the most statistically significant methylation markers. The GTEx web-based visualization tool was used to generate plots while GEO expression data was downloaded and plotted using R. Additionally, R was used to perform and visualize all other analyses.

## Results

### Demographic Comparison of Study Sample

Selected characteristics of the study participants are described by disease status (Table 1). All characteristics are similar across the 4 disease groups with the only significant difference observed for smoking status. There are significantly more ever smokers in the PCa new onset DM group (86%) and significantly more never smokers in the PCa, no DM group (88%). When comparing these characteristics by PCa and non PCa disease groups in the larger Mayo Clinic Pancreatic Cancer resource, we observe that PCa cases are more likely to be male, have a higher usual BMI, more likely to have ever smoked and more likely to be diabetic (Appendix Table A1).

**TABLE 1:** Select characteristics of study participants by disease status.

	Control, new-onset DM (*N* = 7)	Control, no DM (*N* = 7)	PCa, new-onset DM (*N* = 8)	PCa, no DM (*N* = 8)	*p* value
Age	—	—	—	—	0.1394
Mean (SD)	67.6 (3.6)	74.3 (7.2)	66.8 (6.7)	65.6 (10.2)	—
Median	65.0	70.0	64.5	62.5	—
Q1, Q3	65.0, 70.0	69.0, 84.0	61.5, 71.5	58.5, 75.0	—
Range	(65.0–74.0)	(69.0–85.0)	(60.0–79.0)	(52.0–81.0)	—
Sex	—	—	—	—	0.4733
Female	2 (28.6%)	3 (42.9%)	2 (25.0%)	5 (62.5%)	—
Male	5 (71.4%)	4 (57.1%)	6 (75.0%)	3 (37.5%)	—
Race	—	—	—	—	—
White/Caucasian	7 (100.0%)	7 (100.0%)	8 (100.0%)	8 (100.0%)	—
Usual adult BMI	—	—	—	—	0.0953
N	6	6	6	4	—
Mean (SD)	30.0 (3.6)	24.9 (3.6)	29.4 (5.5)	24.0 (5.2)	—
Median	30.9	24.3	29.6	22.4	—
Q1, Q3	27.1, 33.0	22.5, 28.3	25.8, 33.2	20.9, 27.2	—
Range	(24.4–33.7)	(20.5–29.4)	(21.9–36.5)	(19.6–31.6)	—
Smoking status	—	—	—	—	0.0385
Missing	1	1	1	0	—
Never smoker	3 (50.0%)	4 (66.7%)	1 (14.3%)	7 (87.5%)	—
Ever smoker	3 (50.0%)	2 (33.3%)	6 (85.7%)	1 (12.5%)	—
Former smoker	2	2	5	1	—
Current smoker	1	0	1	0	—

*p*-values for continuous variables (age, usual adult BMI) are from an ANOVA F test.

*p*-values for categorical variables are from a Fisher’s Exact test.

### Genome-wide Analysis of CpG Markers and Disease Status

**FIGURE 1:**
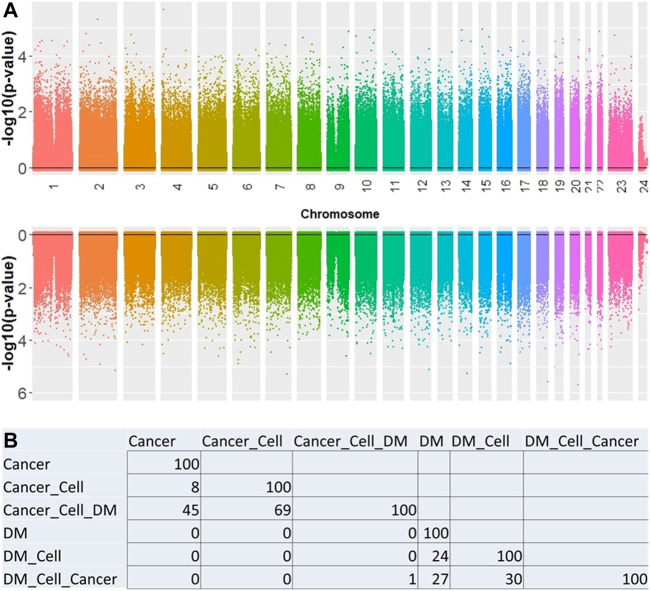
**(A)** Manhattan plot showing the association between CpG sites for cancer (top) and DM (bottom). **(B)** Number of overlapping CpGs significantly associated with the factor of interest between each model. All models adjusted for age and sex. Additional adjustments: Cell = blood cell type; Cancer = PCa; DM = recent onset type 2 diabetes.

Overall, no significant CpG sites were associated with PCa or DM when setting genome-wide significance level to *p*-value < 0.05 × 10^–8^. (Figure 1A). Multiple models were explored in an attempt to understand methylation marker associations in the context of different adjustment factors. The top 100 results of each model were evaluated to identify methylation markers which appeared across multiple models. There was little observed overlap in the sets of top 100 markers (Figure 1B) when comparing cancer to diabetes models.

### Characterization of Top 100 Disease Associated CpG Markers

**FIGURE 2:**
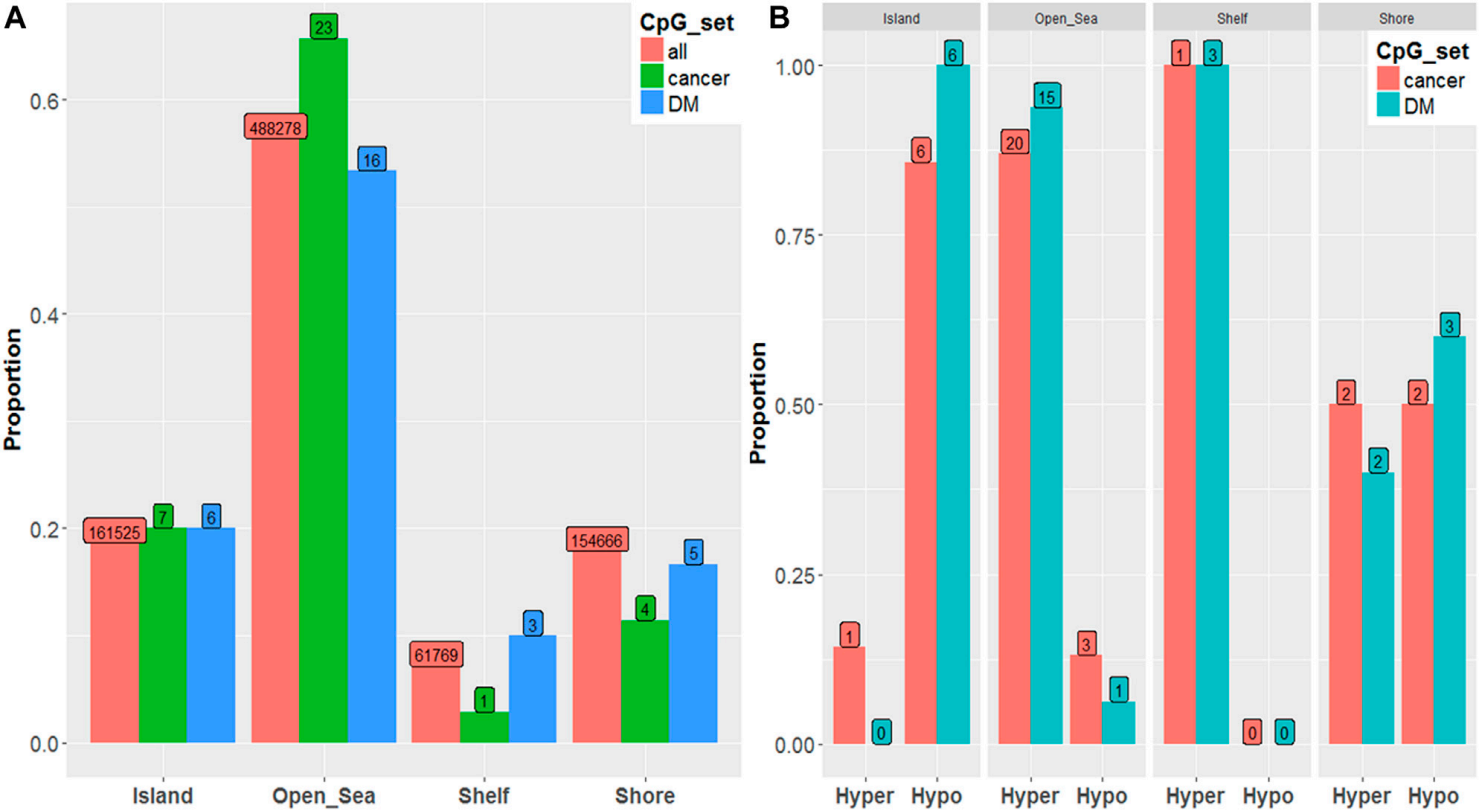
**(A)** Proportion of hypermethylation vs. hypomethylation among significant (*p*-value < 10^–5^) PCa-associated CpGs by genomic region by CpG set. **(B)** Proportion of CpGs residing in each genomic region by CpG set.

We observed a similar hypermethylation/hypomethylation pattern with respect to genomic region. (Figure 2A).When visually comparing all ∼850,000 CpGs to the top 100 disease associated CpGs, (Figure 2B) Open Sea (i.e., CpG sites not classified as island, shore, or shelf) regions are enriched among PCa and there is slight under-representation among the Shore region (i.e., ∼2 kb from islands)..(Illumina, 2016).

**FIGURE 3:**
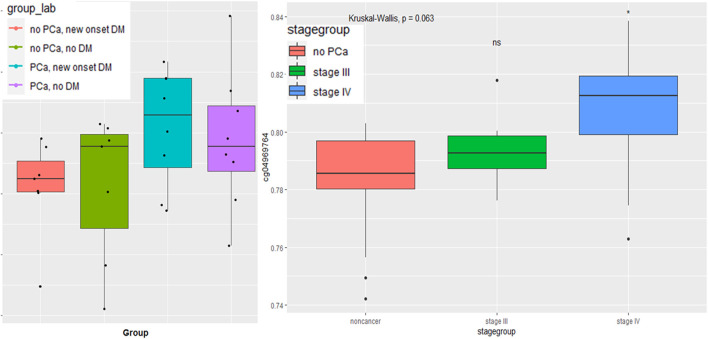
Boxplot of *LAMA5* associated CpG 04969764 methylation **(A)** by PCa and DM disease status and **(B)** by PCa stage.

The CpG marker, CpG04969764, observed in DM and PCa models, shows promise for discriminating patients with recent onset DM with and without PCa and for discriminating later stage PCa from those without PCa. (Figure 3). As illustrated by the boxplot, those participants with both PCa and DM had the highest average methylation of CpG04969764 while those with no PCa but DM had the lowest average methylation. The second boxplot shows that the mean CpG04969764 methylation of stage IV PCa is significantly different than the mean methuylation of the no PCa group.

### Assessment of *LAMA5* Expression and CpG Related Methylation in Publicly Available Datasets

One of the most commonly mentioned and understood biological functions of DNA methylation is related to gene expression. Depending on the gene and the methylation marker, increased methylation (i.e., increased beta value) is frequently either correlated with increased or decreased gene expression. The methylation site is controlling access to that local section of the DNA and therefore influencing the gene expression.

**FIGURE 4:**
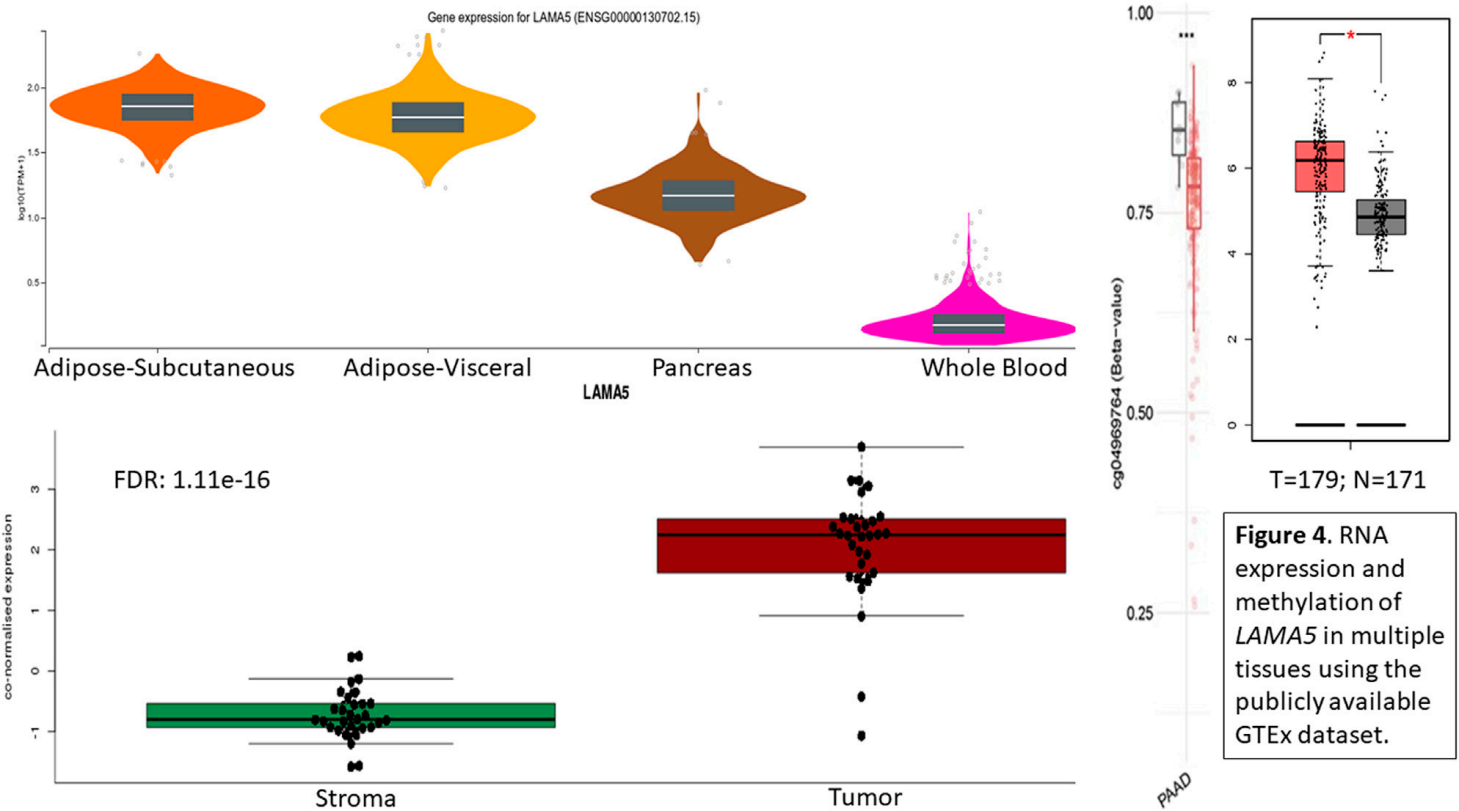
RNA expression and methylation of *LAMA5* in multiple tissues using the publicly available GTEx dataset.

Methylation marker CpG04969764 is located within the *LAMA5* gene region so *LAMA5* gene expression was evaluated in public databases. Using the GTEx database, we can look at the RNA expression levels reported for *LAMA5* across tissue type or specifically in the pancreas or tumor. (Figure 4). When looking at select tissues, we see high expression in adipose tissue, followed by the pancreas, with the lowest in whole blood. Within the pancreas, we see significantly higher expression of *LAMA5* in tumor cells compared with adjacent normal pancreas followed by the lowest expression in stroma cells. It appears that a decrease in methylation leads to increase in *LAMA5* expression. So there is a lower average methylation of cpg markers associated with *LAMA5* and higher average expression of *LAMA5* in pancreatic adenocarcinoma (PAAD) compared to normal tissue.

**FIGURE 5:**
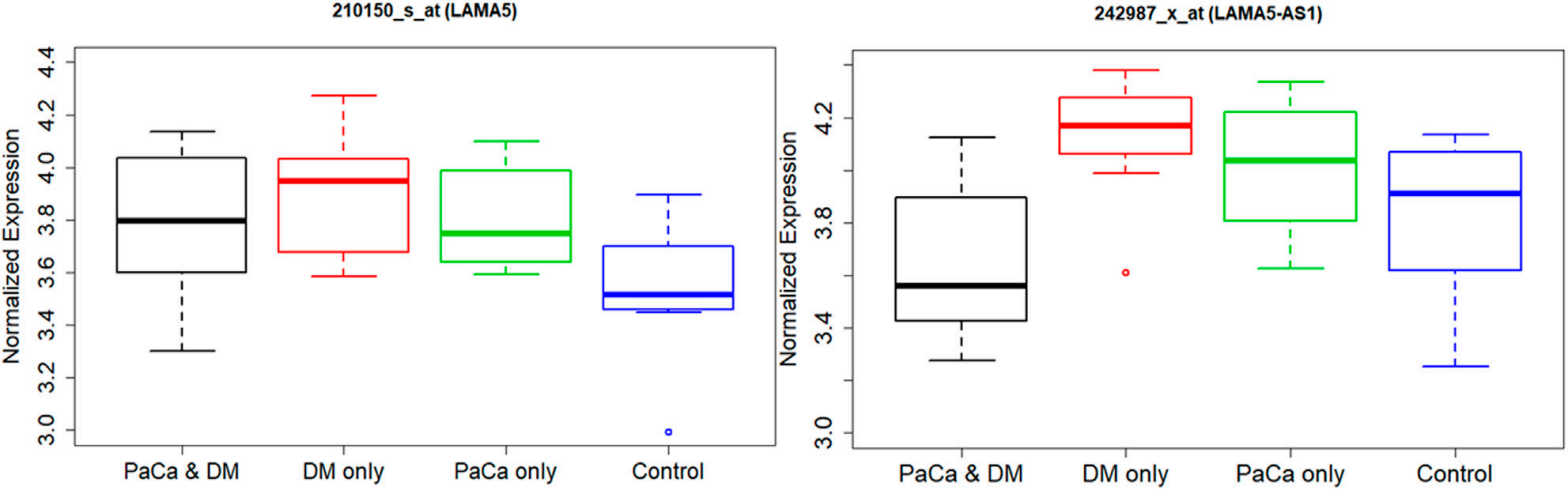
Boxplot of normalized expression of *LAMA5* and *LAMA5-AS*1 by disease status using Gene Expression Omnibus (GEO) data.

Using GEO public database, we evaluated another study for *LAMA5* RNA expression and *LAMA5*-AS1 long non-coding RNA expression (Figure 5). An inverse relationship between methylation of cpg04969764 and *LAMA5* expression is observed in this study. We also see a similar pattern for those with PCa and DM having the lowest expression and those with DM and no PCa having the highest expression. These observed differences are just based on trends with no statistical associations tested.

### Assessment of Methylation-Based Age Estimates

**FIGURE 6:**
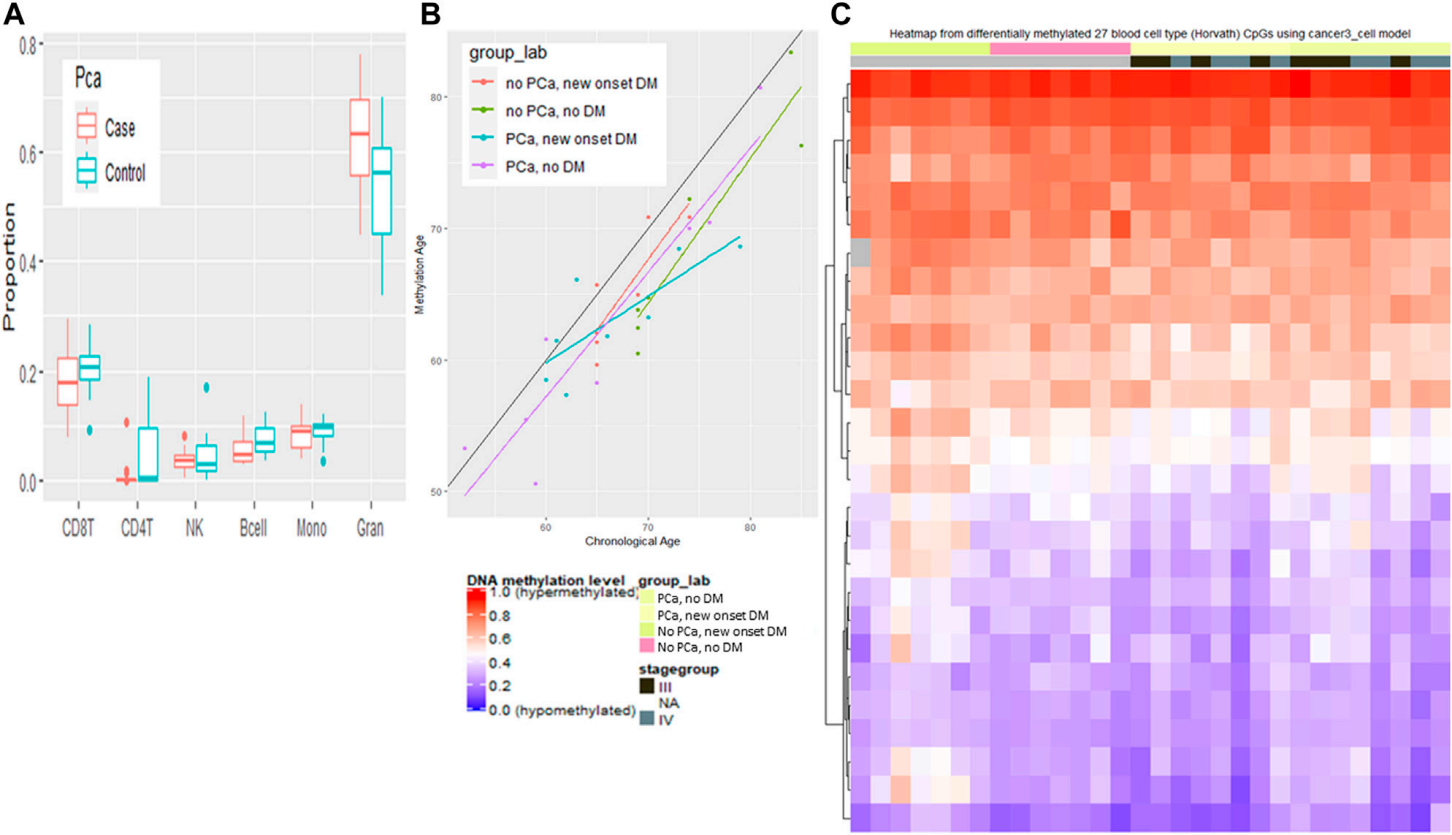
Blood cell type variation among cancer and non cancer groups. **(A)** Proportion of different blood cells types by cancer status. **(B)** Predicated methylation age by chronological age by disease group. **(C)** Heatmap of methylation of 27 Horvath CpGs grouped by cancer stage and disease group.

Each disease group is predicted to have younger methylation-based age estimates compared to their chronological age (Figure 6). We examined associations with age for the 354 CpGs included in the Horvath methylation age predictor (Horvath, 2013). The predicted age based on the calculator showed a strong correlation (*r*) with chronological age among all groups (no PCa, new onset DM *r* = 0.81, PCa, new onset DM *r* = 0.86, PCa, no DM *r* = 0.90, no PCa, no DM *r* = 0.93). The median predicted methylation-based age was younger for all disease groups compared to the chronological age and was 64.9 vs. 65.0 among no PCa, new onset DM, 64.7 vs. 70 among no PCa, no DM, 62.5 vs. 64.5 among PCa, new onset DM, and 59.9 vs. 62.5 among PCa, no DM. The heatmap of the 27/450 Horvath CpGs with a significant difference in methylation shows that there are visually discernible differences to the beta values across the marker set by disease status.

## Discussion

In our pilot study, we did not find any methylation markers significantly associated with PCa or new onset diabetes compared with control the respective control groups. This is not surprising given our small sample size of only 30 and high number of methylation markers of about 850,000. However, this pilot study was designed to provide an exploratory rather than association type of analysis to identify which blood-based methylation markers to investigate in future studies. Therefore, we conducted an exploratory analysis looking at the top 100 (100 lowest *p*-values from our association analysis) methylation markers using either PCa or recent onset DM as the response variable in our regression models. There was 1 methylation marker, CpG 04969764, which appeared in both lists, however, the methylation patterns with respect to hyper-vs. hypo-methylation and genomic region of both 100 marker set were similar. Visually compared to controls with no DM, controls with DM were observed to have a lower median methylation and PCa patients with DM were observed to have higher median methylation at this CpG. In addition, the average methylation was higher in late stage PCa compared to controls. Publicly available data sets including either CpG04969764 methylation or expression of the associated gene, *LAMA5*, support highest methylation among (lower gene expression) among PCa with DM, and the lowest methylation (highest gene expression) among those with no PCa, but with DM.

In our results, we see higher cpg methylation among our PCa with no DM group compared to controls with DM. Seems like PCa has increased methylation and DM reduces methylation. In GTEx we see lower *LAMA5* expression (i.e., higher cpg methylation) in blood compared to pancreas or adipose tissue. *LAMA5* snp associated with higher fasting glucose and higher weight among healthy older adults 65–89. (De Luca et al., 2011). Tumor inflammation induces *LAMA5* expression in colorectal cancer cells. *LAMA5* is required for the successful growth of hepatic metastases where it promotes branching angiogenesis and modulates Notch signalling. (Gordon-Weeks et al., 2019). Deficiency in LN-α5 (i.e., LAMA5) expression resulted in decreased trophoblast proliferation and invasion but increased cell apoptosis, meanwhile, PI3K/AKT/mTOR signaling pathway was impaired by LN-α5 silencing (Zhang et al., 2018). Combining our observations with published literature on *LAMA5* suggests methylation of *LAMA5* maybe a biomarker for those recent onset DM patients with PCa.

Each disease group (PCa with DM, PCa without DM, and DM without PCa, no PCa and no DM) is predicted to have younger methylation-based age estimates compared to their chronological age. The greatest absolute difference was observed among those without PCa and without DM, while the smallest absolute difference among those without PCa but with DM. Another study has observed higher methylation predicted age compared to chronological age in a pooled analysis of prospective cohorts (Chung et al., 2020). Additionally, several studies have observed that accelerated methylation-based age predictors are positively associated with either BMI or obesity (Horvath et al., 2014; Foirito et al., 2017; Nevalainen et al., 2017; Quach et al., 2017).

A strength of the PCa Resource we drew our sample from, is that over 99% of adenocarcinoma cases were confirmed by pathology or medical record. Small sample size and a Caucasian population limit generalization and prevent statistical-based inference beyond this study. This study does not provide any mechanistic details about how age-related changes may influence PCa risk. It is important to note that our methylation analysis was completed using lymphocyte DNA and not pancreatic tumor tissue. Therefore, differential methylation or expression variation of LAMA5 in our study likely represents the response of the body to the presence of disease rather than a modification important to the development of disease.

No blood-based methylation markers were observed to be significantly associated with either PCa or recent onset DM. In our exploratory analysis, one methylation marker, CpG04969764, found in the *LAMA5* gene region was observed in both PCa and DM Top 100 methylation marker sets. Modification of *LAMA5* methylation or *LAMA5* gene function maybe a way to distinguish those recent DM cases with and without PCa, however, additional studies with larger sample sizes and different study types (e.g., cohort) need to be conducted to support this hypothesis.

## Data Availability

The raw de-identified data supporting the conclusion of this article will be made available by the authors, without undue reservation.

## APPENDIX

**TABLE A1:** Select characteristics of participants in the mayo clinic pancreatic cancer resource by disease status.

	Controls (*N*=2,397)	PCA (*N*=3,932)	*p* value
Age at time of pancreatic cancer diagnosis	—	—	0.3935
Mean (SD)	66.5 (8.9)	66.7 (8.7)	—
Median	66.0	67.0	—
Q1, Q3	60.0, 74.0	60.0, 73.0	—
Sex	—	—	<0.0001
Female	1,164 (48.6%)	1,677 (42.7%)	—
Male	1,233 (51.4%)	2,255 (57.3%)	—
Race	—	—	—
White	2,397 (100.0%)	3,932 (100.0%)	—
Usual Adult BMI	—	—	<0.0001
N	2068	3,252	—
Mean (SD)	27.5 (7.7)	28.6 (5.5)	—
Median	26.6	27.8	—
Q1, Q3	24.3, 29.8	24.8, 31.4	—
Smoking Status	—	—	<0.0001
Missing	264	356	—
Never smoker	1,190 (55.7%)	1,662 (46.5%)	—
Ever smoker	943 (44.1%)	1914 (53.5%)	—
Former smoker	879	1,465	—
Current smoker	60	445	—
Self-reported diabetes	—	—	<0.0001
Missing	365	950	—
No	1799 (88.5%)	1954 (65.5%)	—
Yes	233 (11.5%)	1,028 (34.5%)	—

*p*-values for continuous variables (age, usual adult BMI) are from an ANOVA F test.

*p*-values for categorical variables are from a Fisher’s Exact test.
